# Survey on Nursing Home Caregivers’ Basic Knowledge of Oral Health Management: Dental Terminology

**DOI:** 10.3390/dj6030028

**Published:** 2018-07-02

**Authors:** Shizuko Yanagisawa, Masami Yoshioka, Yasuhiko Shirayama

**Affiliations:** 1 Department of Oral Health Science and Social Welfare, Institute of Biomedical Sciences, Tokushima University, Tokushima 770-8504, Japan; shirayama@tokushima-u.ac.jp; 2 Department of Oral Health Sciences, Faculty of Health and Welfare, Tokushima Bunri University, Tokushima 770-8514, Japan; masami@tks.bunri-u.ac.jp

**Keywords:** oral health, dental term, nursing care, caregiver

## Abstract

With the increasing numbers of the elderly requiring care in Japan, the management of their oral health care will require cooperation between medical and dental professionals, and we need to transfer dental knowledge from dental professionals to caregivers. With the help of a questionnaire, we examined 181 caregivers’ depth of understanding regarding 20 typical dental terms with a view to improving the educational instruction provided to them. It was found that except for “clasp”, popular dental terms have largely been accepted. The differences in their degrees of understanding could be owing to the lack of systematic education for caregivers.

## 1. Introduction

In this ultra-aged society with an increasing number of the elderly requiring care in Japan, effective and high-quality nursing care is required. When elderly people reach the stage of requiring nursing care, they are unable to keep their teeth and oral cavities clean, resulting in a quick drop in oral function. It has often been reported that the decline in oral function is closely related to pneumonia [1,2,3,4,5,6], cognitive decline [7,8], decline in the quality of life [9,10,11], and the progress of frailty [12,13]. Oral health management of the elderly requires cooperation between dental professionals and caregivers. As there are few dental professionals in nursing care facilities in Japan [14], caregivers play an important role in managing oral health [11,15]. In order to achieve effective and high-quality oral health care in such facilities, it is necessary to enhance the concerned comprehension and skills of caregivers. The understandings of dental terms are a fundamental issue for the communication between dental professions and caregivers, and it is very important to clarify how extent the caregivers know the dental terms.

Some previous studies have indicated that caregivers have enough knowledge of oral health [16], especially periodontal disease [17], to take care of the elderly. Conversely, it has also been reported that systematic dental education influences the understanding of oral health and diseases [16,18], and some reports suggest that caregivers are unable to assess oral condition and function appropriately [19,20,21,22]. Dental terms used in previous investigations mainly include those about oral health in general; therefore, dental terms regarding the oral health management of the elderly requiring care should be also examined.

While medical and dental professionals need to cooperate to manage oral health care for the elderly, an effective oral health care strategy has not been formulated [14,23]. Thus, we need to pave the way for information transfer from dental professionals to caregivers by enabling the understanding of oral health.

The purpose of this study is to examine caregivers’ depth of understanding of 20 typical dental terms and to improve educational instruction for caregivers through the quantitative assessment of understanding.

## 2. Materials and Methods

### 2.1. Questionnaire Survey

One hundred and ninety-five participants who have worked in eight nursing home facilities participated in the survey. The survey was conducted between January and February 2018 using a mailing method. The questionnaire items included individual attributes (age, sex, experience of caregiver) and understanding of dental terms as shown in Table 1. Twenty dental terms were abstracted from the oral assessment sheet 22 and questionnaire of oral health [24], as has already been reported.

The participants were asked to indicate their depth of understanding of each dental term on a four-point scale: 1 (know it well), 2 (know it a bit), 3 (do not know it well), and 4 (do not know it at all). Higher scores indicated a lower understanding of the concerned dental terms.

### 2.2. Statistical Analysis

The means and standard deviations of scores for each dental term were calculated. Hierarchical cluster analysis using the nearest neighbor method with the Euclidean distance function was conducted to categorize the dental terms according to caregivers’ depth of understanding. All statistical analyses were conducted using SPSS^®^ software (version 24.0, IBM Corp. Armonk, NY, USA).

### 2.3. Ethical Considerations

The present study was approved by the Ethics Committee of Tokushima University Hospital (approval number: 2915). At first, we described the research project and presented the necessary documents to the directors of the nursing facilities and received their consent. Once the study had been explained to the participants and they had been assured of the protection of their personal data, informed consent was obtained and the questionnaire was administered.

## 3. Results

One hundred and eighty-one appropriate answers were received from 195 deliveries, with a response rate of 92.8%. The participants included 51 males (28.2%), 122 females (67.4%), and eight unknown (4.4%). The age ranged from 18 to 70 years with a mean age of 40.6 years and standard deviation (SD) of 13.6 years. The mean years of experience as caregivers was 126.8 months (SD: 82.1 months).

**Figure 1:**
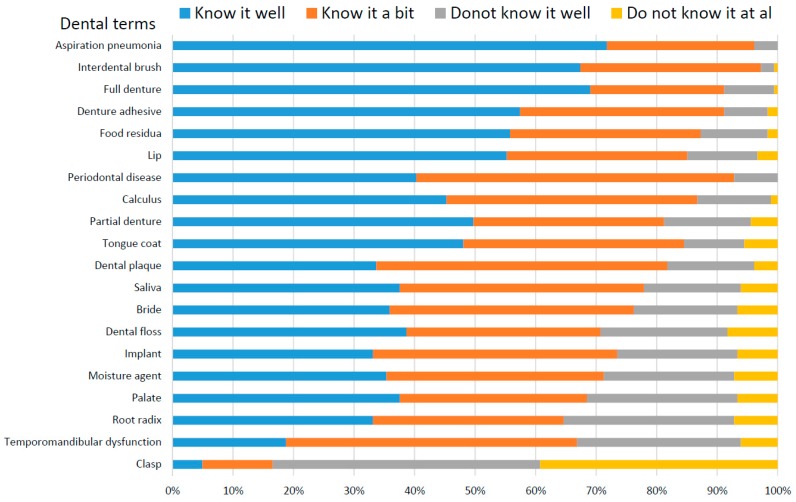
Score distribution of caregivers’ understanding of dental terms.

Figure 1 shows the score distributions on the understanding of dental terms. The score for “aspiration pneumonia” was the lowest (1.32 ± 0.54), after which the scores for “interdental brush”, “complete denture”, “denture adhesive”, and “food residue” increased, in that order. The score of “clasp” was the highest (3.18 ± 0.82), after which the scores for “temporomandibular disorder”, “residual root”, “oral moisture”, “palate”, and “implant” decreased, in that order. The scores for the 19 dental terms except “clasp” were less than 2.3. Almost all caregivers knew the 19 dental terms and all caregivers knew the terms “aspiration pneumonia” and “periodontal disease”.

**Figure 2:**
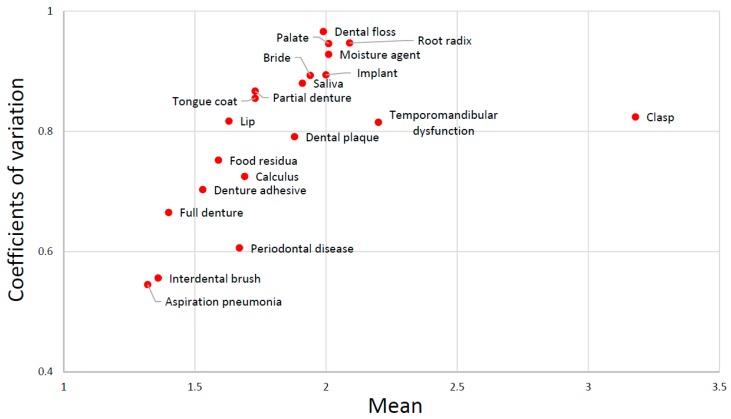
Relationship between the mean and coefficients of variation of the score.

Figure 2 shows the scatter chart between means and coefficients of variation of scores. The means and coefficients of variation of all dental terms except “clasp” were linearly correlated, indicating that it was strikingly different from other terms.

**Figure 3:**
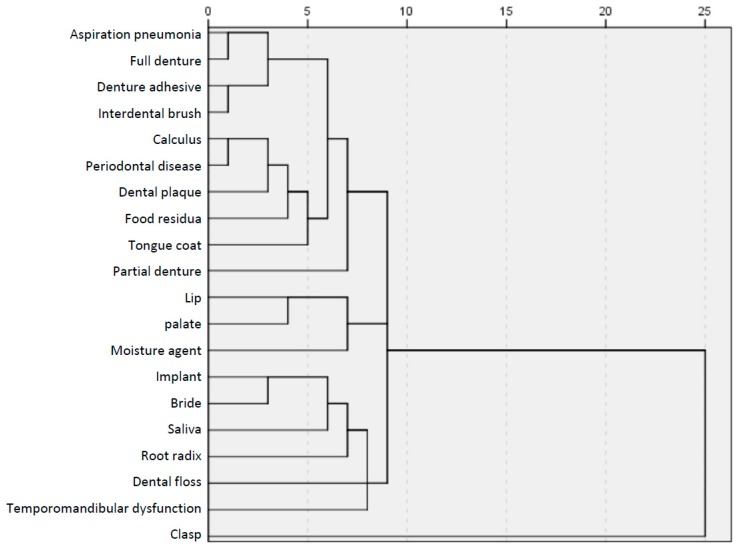
Dendrogram of 20 dental terms by hierarchical cluster analysis.

Figure 3 shows the dendrogram, which is a tree diagram to illustrate the arrangement of clusters produced by hierarchical cluster analysis. The horizontal axis in the dendrogram was labelled distance and refers to a dissimilarity between terms or groups. The horizontal position of the node can be thought of as the dissimilarity between the upper and lower terms/sub-branch clusters. This cluster analysis showed that the 20 terms were categorized into the following groups: well-known terms, including “aspiration pneumonia”, “periodontal disease”, and “complete denture”; unknown terms, including “clasp”; ambiguous terms, including “implant” and “bridge”; and bipolar, including “lip”, “palate”, and “moisture agent”, which were split between the well-known and unknown caregivers.

## 4. Discussion

The results of this study revealed the depth of understanding of dental terms among caregivers in nursing facilities. The results of previous studies were divided as to whether caregivers in Japan have sufficient knowledge of oral health. However, the results of this study demonstrate that caregivers have a baseline knowledge of oral health. Some prosthodontic terms were known, whereas other terms were not known. Some oral health terms also had a similar tendency. The depth and tendency of understanding regarding dental terms are different though the survey. It is speculated that caregivers do not systematically acquire dental knowledge, instead, dealing with dental problems on a case-by-case basis. Essentially, whatever knowledge caregivers have is acquired from the experience of caring for patients. For example, the terms which indicates that they are well known, were “aspiration pneumonia”, “complete and partial denture”, and “denture adhesive”, problems concerning which frequently occur in nursing homes. To put it another way, if you ask a caregiver whether he/she knows the term “clasp”, and he/she knows it, it will be judged that he/she has a good understanding of dental terms. As for self-care instruments of oral health, “interdental brush” had a low score, but the scores for “dental floss” and “moisture agent” were high. It is unclear why the scores for terms that should be equally frequently encountered by caregivers were different. In addition, the absolute values of percentages should be carefully interpreted, because the answering method with four choices might not be well regulated, for example the respondents may had a tendency to select “know it” easily as a response bias.

The lack of understanding of dental terms could be owing to the following factors. The first is a situation in which there is no established educational program for caregivers, and they receive insufficient education on oral health. The second may be because of insufficient response of the dental profession to the nursing facilities. Recently, the oral health service has received preferential remuneration among medical services, but the advantage may be not distributed to all nursing facilities. This may cause the differences in understanding among caregivers. The third may be owing to the depth of observation; caregivers frequently pay attention to major issues like dentures and dental hygiene but miss what lies underneath, like the root radix and clasp.

In conclusion, dental terms, except for clasp, have almost been accepted, and the differences in the degrees of understanding can be owing to the lack of systematic education for caregivers. Therefore, intervention by dental professionals is imperative.

## Figures and Tables

**Table 1:** Questionnaire sheet.

Personal attributes Age: Sex: Male Female Experience of caregiver: years months Do you know the following terms regarding dentistry? Circle the relevant number.				
	**Know It Well**	**Know It a Bit**	**Don’t Know It Well**	**Don’t Know It at All**
	**1**	**2**	**3**	**4**
Calculus	1	2	3	4
Dental plaque	1	2	3	4
Food residua	1	2	3	4
Periodontal disease	1	2	3	4
Temporomandibular dysfunction	1	2	3	4
Aspiration pneumonia	1	2	3	4
Residual root	1	2	3	4
Tongue coat	1	2	3	4
Implant	1	2	3	4
Bride	1	2	3	4
Full denture	1	2	3	4
Partial denture	1	2	3	4
Clasp	1	2	3	4
Lip	1	2	3	4
Palate	1	2	3	4
Saliva	1	2	3	4
Denture adhesive	1	2	3	4
Interdental brush	1	2	3	4
Dental floss	1	2	3	4
Moisture agent	1	2	3	4
